# Information and communication technology use by students with disabilities in higher education during the COVID-19 pandemic

**DOI:** 10.1007/s10209-023-00997-w

**Published:** 2023-05-10

**Authors:** Hayato Kishira, Ginga Sasaki

**Affiliations:** 1 Graduate School of Comprehensive Human Sciences, Degree Programs in Comprehensive Human Sciences, Master’s Program in Disability Sciences, University of Tsukuba, 1-1-1, Tennodai, Tsukuba-Shi, Ibaraki, 305-8572 Japan; 2 Institute of Human Sciences, University of Tsukuba, 1-1-1, Tennodai, Tsukuba-Shi, Ibaraki, 305-8572 Japan

**Keywords:** Disabilities, Information and communication technology, COVID-19, Distance learning, College students

## Abstract

Recently, due to the spread of COVID-19, the implementation of remote learning has been increasing worldwide. This study aims to analyze the difficulties and convenience of using information and communication technology (ICT) for students with disabilities and changes in their perceptions of ICT use after the completion of courses for each form of remote learning. The survey included 122 students with disabilities and 314 students without disabilities via a web-based questionnaire. The questionnaire consisted of four situations, categorized according to the type of remote classes. We conducted an analysis of variance using a two-factor mixed design with disability (non-paired: two levels) × situations (paired: four levels) regarding perceptions of resistance toward ICT and self-rated comprehension as the dependent variables. Results show that students with disabilities were more positive about using ICT than students without disabilities in many items. However, before the courses that required the use of relatively new application software, such as web conferencing systems, students with disabilities showed significantly higher levels of resistance and lower levels of self-assessed comprehension. Further, a comparison of the amount of change in perceptions before and after the course reveals that students with disabilities showed significantly more improvement in negative items before the course. These results suggest the importance of providing opportunities for students with disability to learn how to use ICT and understand its convenience in an environment similar to an actual classroom, given the rapid changes occurring in ICT.

## Introduction

### COVID-19 pandemic and the expansion of remote learning

In recent years, the implementation of distance education has increased worldwide [1]. According to the National Center for Education Statistics (NCES), approximately 37.2% of students enrolled in U.S. higher education institutions in FY 2019 were in distance education. Comparisons with historical statistics from the NCES show a gradual increase since FY 2012, with an overall increase of approximately 10.8% [2]. The advantages of distance education include the ability of students to access classes from anywhere and the instructor’s flexibility to offer classes at their convenience [1]. In contrast, the implementation of distance education in Japanese higher education has lagged far behind. Regarding the percentage of four-year public universities offering distance education, Japan had 13.3% in 2006 [3], compared to 89.0% in the U.S. in the same year [4]. However, distance education, which has received attention primarily in addressing diverse needs, has been promoted internationally as emergency remote learning to prevent the spread of COVID-19 (coronavirus disease 2019) since 2020. In China, for example, the “Disrupted classes, undisrupted learning” initiative provided flexible remote learning to more than 270 million students [5]. Ali [6] also investigated the promotion of remote learning in various countries in the wake of the COVID-19 outbreak. He emphasized the case of Italy, the first EU member state to close its universities and shift classes online, and the successful rapid deployment of remote learning by New York University, Shanghai, and Duke Kunshan University using Zoom, Moodle, and other tools. Japanese higher education institutions have also been required to ensure learning opportunities using various media, and classes at higher education institutions have been primarily conducted remotely [7]. Of the higher education institutions offering classes as of July 1, 2020, the total percentage of respondents who indicated that the type of their classes was “a combination of face-to-face and remote classes” or “remote classes” was approximately 83.8% [8].

### Remote learning and information and communication technology

Information and Communication Technology (ICT) plays an essential role in implementing remote learning. UNESCO [9], in its “Information and Communication Technologies in Teacher Education, A Planning Guide,” states, “ICT is a scientific, technological and engineering discipline and management technique used in handling information, its application and association with social, economic and cultural matters” [10]. However, ICTs are challenging to describe accurately because the types and nature of ICTs change rapidly [11]. Therefore, when researching ICTs, we should clarify their characteristics in a specific context [12]. There is considerable helpful information on the parts of ICT used in remote learning. For example, Fichten et al. [13] surveyed the use of mobile devices (smartphones and tablets) and application software to continue learning during the COVID-19 pandemic. The results showed that, for mobile devices, smartphones were more commonly used than tablets for accessing videoconferencing systems, word processing software, application software related to collaboration and document sharing, and university-specific portals sites. Examples of application software include Zoom, Microsoft Teams, Microsoft Word, Google Document, OneDrive, Dropbox, StudiUM, and Omnivox [13].

### Students with disabilities in Japanese higher education institutions

Since 2005, JASSO has conducted an annual survey of the number of students with disabilities enrolled in Japanese higher education institutions and the support available to them. The term “students with disabilities” in JASSO’s survey refers to students who meet one of the following conditions [14]:

- Students with a physical disability certificate or a mental disability certificate or an intellectual disability certificate;
- Students who are identified as having a disability at a health check.

For the 2021 survey, JASSO [14] collected data from 1176 institutions. The result showed that the number of students with disabilities enrolled in Japanese higher education institutions in FY2021 was 40,744, or 1.26% of the total student population. Additionally, it provided information on specific support offerings as the following. First, support in the class includes “providing an accommodation letter” (575 institutions), “seating arrangements” (561 institutions) and “attendance arrangements” (521 institutions) Second, support outside of class included “professional counseling” (472 institutions), “guidance on self-management” (317 institutions), and “cooperation with medical institutions” (300 institutions)”.

However, there are two problems with the number of students with disabilities enrolled in Japanese higher education institutions. First, as in other countries, the Japanese office for students with disabilities does not fully grasp the number of students with disabilities who do not disclose their disabilities to educational institutions [15]. In other words, the term “students with disabilities” in JASSO’s survey refers only to students registered with the office for students with disabilities. Second, as indicated by JASSO, the percentage of students with disabilities enrolled in Japanese higher education institutions is significantly lower than that of students with disabilities indicated by other countries [16]. One cause of this issue has been attributed to the lack of deployment of the office for students with disabilities. Specifically, for the 2021 survey, only 292 out of 1,176 institutions have a dedicated office for students with disabilities [14]. That said, recognition of support for students with disabilities in Japan has increased since the passage of the Act on the Elimination of Disability Discrimination in June 2013, and it is expected to continue to develop in the future [16].

### ICT use by students with disabilities in higher education institutions

The number of students with disabilities enrolled in higher education institutions is rising. For example, in Ireland, the percentage of students with disabilities enrolled in higher education institutions’ disability support services has steadily increased, reaching 6.3% of the total student population in FY 2019 [17]. In the same year, the percentage of students with disabilities in Japan was 1.17%, an increase of 3835 from the previous year [18]. Therefore, many students with disabilities enrolled in higher education institutions must use ICT in distance classes [19]. Students with disabilities in higher education hold numerous positive opinions about using ICT [20, 21]. A specific example of the usefulness of ICT for students with disabilities is the multiple benefits gained by simply providing online materials created in Microsoft PowerPoint before class. Specifically, giving presentation materials in advance serves as a support for students with learning disabilities who have difficulty following slides with their eyes during the course or as a backup for students with mental illness or chronic health issues when they are absent [22]. It is not only in higher education that the use of ICT during learning for students with disabilities is considered to play an important role. The use of ICT for students with disabilities in primary and secondary education has been shown to increase the effectiveness of instruction when information equipment is tailored to their needs [23]. Effective ICT use by students with disabilities will become increasingly important in various educational settings.

However, Seale et al. [24] argue that while students with disabilities can undoubtedly benefit from ICTs, they may also be disadvantaged in various aspects. To illustrate this, it has been pointed out that visually impaired students who use screen readers need particular operation practice when using the web conferencing system and Learning Management System (LMS). The subtitle information used by deaf students is incorrectly converted when using the result of the speech recognition system without modification [25]. Therefore, while there are many possibilities for introducing ICT into the educational environment for students with disabilities, there are also numerous unsettling aspects [26].

### Classification of remote learning

JASSO [25] surveyed university accommodations for students with disabilities. The survey also asked students to rate the ease of taking remote learning compared to face-to-face courses. In doing so, it categorized the remote classes into “synchronous remote learning”, “asynchronous remote learning with streaming video” and “asynchronous remote learning without streaming video”. Synchronous remote learning progresses in real-time using a web conferencing system. Asynchronous remote learning with streaming video refers to lecture videos uploaded to video streaming services such as Microsoft Stream and YouTube. In contrast, asynchronous remote learning without streaming video refers to classes in which non-video materials are distributed online [25]. The results showed that students with disabilities found all types of classes easier to attend than face-to-face classes on average. Furthermore, only synchronous remote learning received a relatively low rating, suggesting differences in the ease of taking each remote learning. Further studies comparing ICT use by students with disabilities by class type include an international comparison of face-to-face and online course models by Heiman et al. [19]. Heiman et al. [19] compared the types of ICTs used and perceptions of accessibility of the ICTs between students enrolled in the traditional face-to-face teaching model (in Canada) and students enrolled in the distance teaching and learning model (in Israel). The results showed that students attending distance learning classes have more knowledge and access to ICTs than students attending the traditional face-to-face teaching model. However, we have not found any studies that have statistically compared different types of distance learning.

### Purpose

While the COVID-19 pandemic is an emergency, it is also an excellent opportunity for rapid growth through the use of many ICT-based educational methods. Ali [6] highlighted that ICT is a powerful force transforming educational settings worldwide and that the pandemic provides an opportunity to promote distance education using ICT. In particular, investigating the use of ICT by students with and without disabilities in higher education and identifying its benefits and current problems would be of great value in realizing a better educational environment in the future. However, to the best of our knowledge, not enough research has been conducted focusing on ICT use by students with disabilities, and comparisons with students without disabilities have not been made. In addition, no study has been performed worldwide to clarify the difficulties and convenience of ICT use for each type of distance learning by classifying them as synchronous remote learning or asynchronous remote learning. Therefore, this study investigated ICT use in remote learning for students with and without disabilities by focusing on the following three research questions.

- RQ1: What are the characteristics of the ICT used by students with or without disabilities?
- RQ2: How do students with disabilities perceive the difficulty and convenience of each type of remote learning compared to students without disabilities?
- RQ3: How do their perceptions of application software change before and after the course compared to students without disabilities?

This study aimed to use the results obtained to determine what is required to realize better ICT use during learning by students with disabilities. ICT objects in this study included:

- Device equipment used during learning (smartphones, laptops, desktop PCs, and tablets);
- Application software like LMS used for attending lectures and creating and managing assignment submissions.

## Methods

### Participants and survey period

The study included students with and without disabilities enrolled in Japanese universities. The survey period was from July 1, 2021, to October 15, 2021. Responses from students with disabilities and students without disabilities were collected using different procedures. For students with disabilities, we asked the Disability Resource Center at 103 universities (29 national universities, 14 public universities, and 60 private universities). Finally, survey cooperation was obtained from 15 national, 4 public, and 17 private universities (36 in total) (survey cooperation rate: 35.0%). Each university sent survey forms to students with disabilities. Data with missing responses regarding grade, disability, or class enrollment status, or those with multiple answers were excluded. A total of 122 students with disabilities were eligible for the program. In addition, for students without disabilities, we requested 560 college students nationwide to fill in the survey using a survey panel from an internet research company, resulting in a final sample of 314 students without disabilities (56.1% response rate).

### Survey contents

We asked students with disabilities a total of 90 questions, and students without disabilities a total of 80 questions. The questionnaire was mainly composed of the sections listed in 2.2.1 through 2.2.3. For devices and application software, the classification by Fichten et al. [27] was used as a reference. Specifically, the questions were designed as ICTs of different natures: general ICTs (e.g., Microsoft Word and e-mail programs) and adaptive ICTs for students with disabilities (e.g., alternative input devices and screen readers). In this study, among ICTs with adaptive features, application software used by installing it on a device was considered “adaptive application software”, and the rest were considered “adaptive devices”, In the survey contents for students without disabilities, questions regarding the type of disability and the use of adaptive ICTs were removed.

#### Fact Sheet

The respondents were asked to provide information regarding their grades, age, affiliation, course enrollment, and disabilities. Only students with disabilities were asked to respond about the type of disability.

#### Devices

Respondents were asked to indicate their preference for device operation using a five-point Likert scale ranging from “1: I do not like it at all” to “5: I like it very much”. The same responses were requested for self-assessed proficiency in device operation. In addition, the survey included an item asking whether there was any device that the students felt they wanted to use during the learning process but were unable to do so. Only students with disabilities were asked to respond about the use of adaptive devices.

- Preference for device operation: The higher scores indicate that respondents like to operate devices.
- Self-assessed proficiency in device operation: The higher scores indicate that respondents consider themself good at operating devices.

#### Application software

The items were created drawing on JASSO [25], assuming four situations: three class types (synchronous remote learning, asynchronous remote learning with streaming video, and asynchronous remote learning without streaming video) and assignment preparation and submission. Specifically, the survey was composed of items that asked about the frequency of device use and application software used in each situation and the degree to which the respondents found ICT use “difficulty” and “convenience” on a five-point Likert scale (1 = strongly disagree, 5 = strongly agree). In addition, the survey included a five-point Likert scale (1 = strongly disagree, 5 = strongly agree) on the perception of ICT use before and after attending courses for four items: “I think it is easy to use”, “I think it is useful”, “I have some resistance to using it” and “I understand how to use it well”. Only students with disabilities were asked to indicate their use of adaptive application software. Among the questions, the types of devices used for learning (smartphones, laptops, desktop computers, and tablets) and the types of application software used in synchronous remote learning were developed based on the questions used in the Toyo University Institute of Social Science ICT Education Research Project [28]. Among the survey items in this study, the following four items were analyzed according to the research objectives:

- Respondents find using ICT in that course type more “difficulty”, as indicated by higher scores in difficulty in using ICT;
- Respondents find using ICT in that course type more “convenience”, as indicated by higher scores in convenience in using ICT;
- Respondents have more resistance to ICT used in that course, as indicated by higher scores in the statement “I have some resistance to using it”;
- Respondents have a higher level of self-assessed comprehension regarding using ICT in the course, as indicated by higher scores in the statement “I understand how to use it well”.

### Analysis method

IBM SPSS Statistics (ver. 28.0) was used for statistical analysis. Kolmogorov–Smirnov normality tests and Shapiro–Wilk tests were performed for each questionnaire item. The significance level for both tests was 5%. Differences between students with disabilities and those without disabilities in the perception of difficulty, convenience, and resistance to ICT use in remote classes were compared by analysis of variance in a two-factor mixed design with disability (no-paired: 2 levels) × situations (paired: 4 levels) as the independent variables. By adopting a two-factor mixed design, we aimed to examine the perceptions of ICT among students with disabilities in specific course types. In adopting a two-factor mixed design, the analysis was limited to students who answered all four situations. In addition, Pearson’s chi-square test and residual analysis were performed on the responses of students with disabilities × students without disabilities regarding using the application software.

### Ethical considerations

The study was approved by the Research Ethics Committee, Faculty of Human Sciences, University of Tsukuba, Japan (Proposal No. 2021-62A). In the survey, we explained that respondents would not be disadvantaged, that cooperation in the research was voluntary, that the survey results would be made public in a form that did not identify individuals, and that responses to the survey would be considered consent to participate in the study.

## Results

### Descriptive statistics on participants’ attributes and ICT use

The mean age ± standard deviation was 20.5 ± 2.01 years for students with disabilities and 20.4 ± 2.72 years for students without disabilities. Table 1 shows the disability type, type of university, and the number of university students. The most common disability type among students with disabilities was “mental health condition,” with 62 (50.8%) students, while 46 (37.7%) students with disabilities had multiple disabilities. The most common type of university was “private university,” with 78 (63.9%) students with disabilities and 209 (66.6%) students without disabilities. The most common college size was “10,001 students or more,” with 54 (44.3%) students with disabilities and 127 (40.4%) students without disabilities. The grades of the participants are shown in Table 2. A χ^2^ test by disability × grade and a residual analysis showed no difference in grade between students with and without disabilities (χ^2^ = 2.277, *df* = 3, *n.s.*). Regarding the classification of the participants based on their undergraduate program, social sciences were the most common for both students with and without disabilities, with 36 (29.5%) and 115 (36.6%) participants, respectively. The participants’ most common class attendance status, both for students with disabilities and without disabilities, was “attended both in FY2020 and FY2021”,.

**Table 1:** Basic information of the participants

			Students with disabilities	Students without disabilities
			N (%)	N (%)
Type of disability				
Visually impaired			8 (6.6)	
Hearing impaired			15 (12.3)	
Physically impaired			18 (14.8)	
Chronic medical condition			17 (13.7)	
Specific Learning Disorder (SLD)			5 (4.0)	
Attention Deficit Hyperactivity Disorder (ADHD)			23 (18.5)	
Autism Spectrum Disorder (ASD)			32 (25.8)	
Mental health condition			62 (50.8)	
Others			4 (3.2)	
Without disability				314 (100.0)
Type of university				
National universities			37 (30.3)	76 (24.2)
Public universities			7 (5.7)	28 (8.9)
Private universities			78 (63.9)	209 (66.6)
N/A			0 (0.0)	1 (0.3)
Number of students in the university				
10,001	–		54 (44.3)	127 (40.4)
5,001	–	10,000	24 (19.7)	49 (15.6)
1,001	–	5000	36 (29.5)	79 (25.2)
1,000	–		4 (3.3)	56 (17.8)
N/A			4 (3.3)	3 (1.0)

% represents the composition in each group

**Table 2:** Participants’ grades

Grade	Students with disabilities	Students without disabilities
	N (%)	N (%)
1st year	34 (27.9)	77 (24.5)
2nd year	33 (27.0)	79 (25.2)
3rd year	22 (18.0)	78 (24.8)
4th year	31 (25.4)	80 (25.5)
Others	2 (1.6)	0 (0.0)
Total	122 (100.0)	314 (100.0)

The most commonly-used LMS was Moodle, with 101 students (23.4%). This was followed by Google Classroom (90 students: 20.9%), Manaba (59 students: 13.7%), and Web Class (33 students: 7.7%). Among adaptive devices, 25 (20.5%) respondents selected “headphones/earphones”, followed by 10 (8.2%) who selected “microphone”, and eight (6.6%) who selected “smartpen”. The most common response regarding the adaptive devices was “nothing in particular”, with 70 respondents (57.4%). In terms of the use of adaptive application software, 10 respondents (8.2%) used “speech recognition systems (e.g., UDtalk)”, six respondents (4.9%) used “software for scanners and OCR (e.g., Microsoft Office Lens, e.Typist)”, and four respondents (3.3%) used “software related to note-taking (e.g., IPtalk, IPtalk Viewer)”. The most common response regarding the adaptive application software was “nothing in particular” with 81 respondents (66.4%). The disability types of students who used adaptive technologies are shown in Table 3.

**Table 3:** Disability types of students who used adaptive technologies

Type of disability	Adaptive devices		Adaptive application software	
	Used	Not used	Used	Not used
Visually impaired	5	4	5	2
Hearing impaired	9	9	10	4
Physically impaired	4	10	3	15
Chronic medical condition	7	9	0	14
Specific Learning Disorder (SLD)	5	2	4	0
Attention Deficit Hyperactivity Disorder (ADHD)	14	15	7	12
Autism Spectrum Disorder (ASD)	14	22	6	21
Mental health condition	15	37	9	46
Others	1	3	0	3

### Comparison of ICT use and preferences by disability status (RQ1)

A two-factor mixed design was used for the frequency of device use, with disability × situations as the independent variable, except for the “do not own” response. Results showed that the main effect of disability was not significant for any device. In contrast, only the main effect of situations on the frequency of laptop use was significant (*F* (1, 250) = 4.269, *p* < 0.05). In addition, multiple comparisons by Fisher’s Least Significant Difference (LSD) showed that the frequency of use in asynchronous remote learning without streaming video was significantly lower than in asynchronous remote learning with streaming video (*p* < 0.05) and assignment preparation and submission (*p* < 0.001). Hence, the type of device used was the same regardless of the disability.

For each of the four situations, a χ^2^ test and residual analysis were conducted with each application software use (2 levels) × disability (2 levels). Asynchronous remote learning without streaming video showed significantly higher results for students with disabilities for the use of Microsoft Word (χ^2^ (1) = 12.98, *p* < 0.001) and Microsoft PowerPoint (χ^2^ (1) = 8.86, *p* < 0.01) than for students without disabilities. Furthermore, considering assignment preparation and submission, the use of Microsoft Word (χ^2^ (1) = 15.99, *p* < 0.001) and Microsoft PowerPoint (χ^2^ (1) = 4.03, *p* < 0.05) was significantly higher for students with disabilities. Other application software such as Notepad (application software) (χ^2^ (1) = 15.82, *p* < 0.001) and Sticky Notes (application software) (χ^2^ (1) = 5.59, *p* < 0.05) also showed significantly higher use for students with disabilities. Thirteen students with disabilities listed Microsoft Word and Microsoft PowerPoint as the “application software that they often found particularly useful” or “ICT that they would like to continue using in the future”. Five respondents cited “familiarity” and two cited “ease of editing” when referring to the reasons.

Regarding preferences for device operation, responses belonging to the mean + 1 SD were grouped into a “like group”, whereas responses belonging to the mean -1 SD were grouped into a “do not like group”, resulting in a two-level preference. Next, a *χ*^2^ test and residual analysis were performed by disability (2 levels) × preference (2 levels). Finally, the same procedure was used to analyze self-rated proficiency in device operation. The results showed that compared to students without disabilities, students with disabilities had significantly more individuals in the “like group” (*p* < 0.01) and the “good at it group” (*p* < 0.05). Hence, students with disabilities were more favorable to the use of ICT than students without disabilities. Additionally, students with disabilities tended to prefer application software that they were familiar.

### Comparison of difficulty and convenience of using ICT with and without disabilities

#### Difficulty and convenience of using ICT (RQ2)

A two-factor mixed design of disability × situations was used with the degree of perceived “difficulty” and “convenience” as a dependent variable to determine students’ degrees of difficulty and convenience. First, using the degree of “difficulty” as the dependent variable, the main effect of disability status was found to be significant (*F* (1, 258) = 16.631, *p* < 0.001) (Table 4). This result showed that students with disabilities found using ICT in distance classes significantly less difficulty than students without disabilities. Furthermore, the main effect of situations was also significant (*F* (3, 804) = 30conve.965, *p* < 0.001). No significant difference was found for the disability × situations interaction (*F* (3, 804) = 1.875, *n.s.*). Considering there were no significant differences in the interactions, multiple comparisons by Fisher’s LSD were performed for the main effects of situations. The results showed that the mean of the degree of “difficulty” in synchronous remote learning was significantly higher than the mean in the other three situations (*p* < 0.001). Hence, students with disabilities experienced less difficulty using ICT than those without disabilities. Furthermore, the most challenging course type regarding ICT use for all students, regardless of disability, was synchronous remote learning.

**Table 4:** Comparison of the difficulty and convenience of using ICT for students with and without disability

Item	Disabilities	*N*	*M*	*SD*	*p*	
Degree of “difficulty”	With	75	2.240	1.320	< .001	***
	Without	195	2.715	1.111		
Degree of “convenience”	With	74	3.926	1.009	< .001	***
	Without	195	3.415	0.965		

With the degree of “difficulty”, higher scores indicate more significant difficulties students perceive in ICT use. With the degree of “convenience”, higher scores indicate more conveniences students perceive in ICT use

Next, using the degree of perceived “convenience” as the dependent variable, the main effect of disability status was found to be significant (*F* (1, 267) = 26.579, *p* < 0.001) (Table 4). This result showed that students with disabilities perceived ICT use in remote learning to be significantly more “convenience” than students without disabilities. Furthermore, the main effect of situations was also significant (*F* (3, 801) = 13.464, *p* < 0.001). No significant difference was found for the disability × situations interaction (*F* (3, 801) = 1.767, *n.s.*). Because there were no significant differences in the interactions, multiple comparisons by Fisher’s LSD were performed for the main effects of situations. The results showed that the mean of the degree of “convenience” in asynchronous remote learning with streaming video was significantly higher than in synchronous remote learning (*p* < 0.05), asynchronous remote learning without streaming video (*p* < 0.001), and assignment preparation and submission (*p* < 0.001). The mean in synchronous remote learning was significantly higher (*p* < 0.05) than in asynchronous remote learning without streaming video (*p* < 0.01) and assignment preparation and submission (*p* < 0.001). Hence, students with disabilities found ICT more convenient than students without disabilities. Furthermore, the most convenient course type regarding ICT use for all students, regardless of disability, were synchronous remote learning and asynchronous remote learning with streaming video.

#### The amount of change in perceptions of the application software before and after the course (RQ3)

Based on the retrospective evaluation, “perception after attending the course”—“perception before attending the course” was used as the amount of change in perception. A two-factor mixed design of disability × situations was used with the amount of change as the dependent variable to determine how the four perceptions toward the application software depended on the disability and situations. The four perceptions of application software are “I think it is easy to use,” “I think it is useful”, “I have some resistance to using it” (resistance), and “I understand how to use it well” (self-assessed comprehension).

**Fig. 1:**
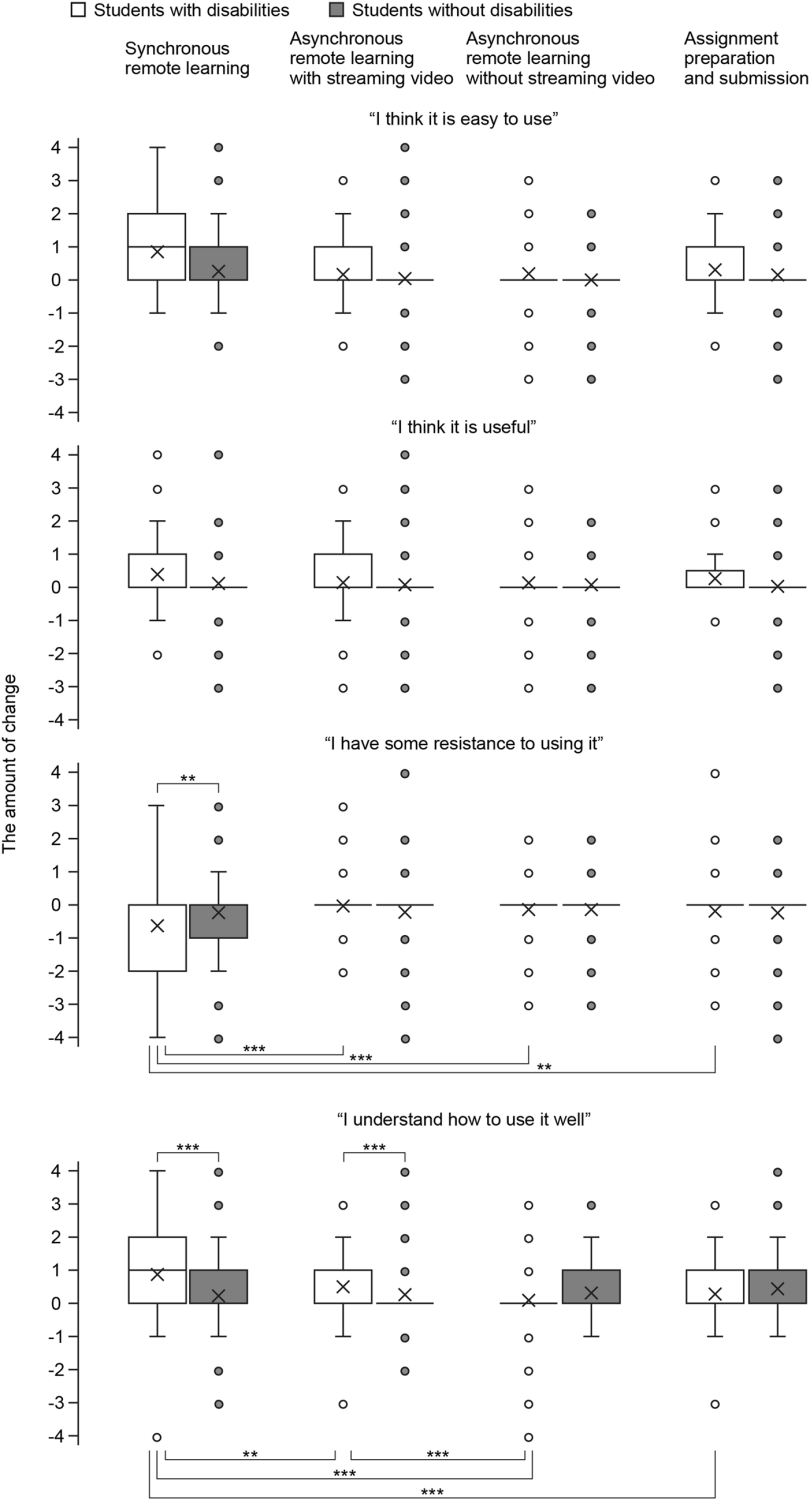
Comparison of the amount of change in perceptions of the use of application software

The results are shown in Fig. 1. First, we tested for the simple main effect of situations regarding the amount of change in the perception of resistance. The results showed that the simple main effect of situations for students with disabilities was significant (*F* (3, 639) = 6.89, *p* < 0.001). However, there was no significant difference in the simple main effect of situations for students without disabilities (*F* (3, 639) = 0.28, *n.s.*). Multiple comparisons using Bonferroni’s method showed significantly more decrease in the disabled students’ perception of resistance in synchronous remote learning than in in asynchronous remote learning with streaming video (*p* < 0.001), asynchronous remote learning without streaming video (*p* < 0.001), and assignment preparation and submission (*p* < 0.01). Next, we tested for a simple main effect of disability. Results showed that the simple main effects of disability in synchronous remote learning were significant (*F* (1, 225) = 7.44, *p* < 0.01). A comparison of the means showed that the score decrease in resistance in synchronous remote learning for students with disabilities was more significant than for students without disabilities. Hence, when students with disabilities experienced taking synchronous remote learning, their resistance to ICT used in such courses decreased dramatically. This tendency was unique to students with disabilities and not to students without disabilities.

The change in the perception of self-assessed comprehension was also tested with a simple main effect test for situations. The results showed that the simple main effect of situations for students with disabilities was significant (*F* (3, 675) = 13.85, *p* < 0.001); however, there was no significant difference in the simple main effect of situations for students without disabilities (*F* (3, 675) = 1.89, *n.s.*). Multiple comparisons showed significantly more positive changes in the disabled students’ perception of self-assessed comprehension in synchronous remote learning than in asynchronous remote learning with streaming video (*p* < 0.01), asynchronous remote learning without streaming video (*p* < 0.001), and assignment preparation and submission (*p* < 0.001). In addition, there was a significantly more positive change in asynchronous remote learning with streaming video for students with disabilities than in asynchronous remote learning without streaming video (*p* < 0.001). Next, we tested for a simple main effect of disability. The results showed a significant simple main effect of disability status in synchronous remote learning (*F* (1, 225) = 18.17, *p* < 0.001) and a significant simple main effect of disability status in asynchronous remote learning with streaming video. A comparison of means showed that the positive change in synchronous remote learning and asynchronous remote learning with streaming video was more significant for students with disabilities than those without disabilities. Hence, when students with disabilities experienced synchronous remote learning or asynchronous remote learning with streaming video, their self-assessed comprehension of the ICT used in such courses increased dramatically. This trend was unique to students with disabilities and not to students without disabilities.

#### Pre-course perception of application software use

Because there were significant differences in the amount of change in recognition by disability and situations, it was decided to make comparisons for pre-class perceptions. We aimed to examine the nature of the change in salient perceptions. More specifically, we checked whether the significant difference in perceptions was a catch-up of perceptions that were particularly negative before the class or a positive change among perceptions that were all initially at the same level.

**Fig. 2:**
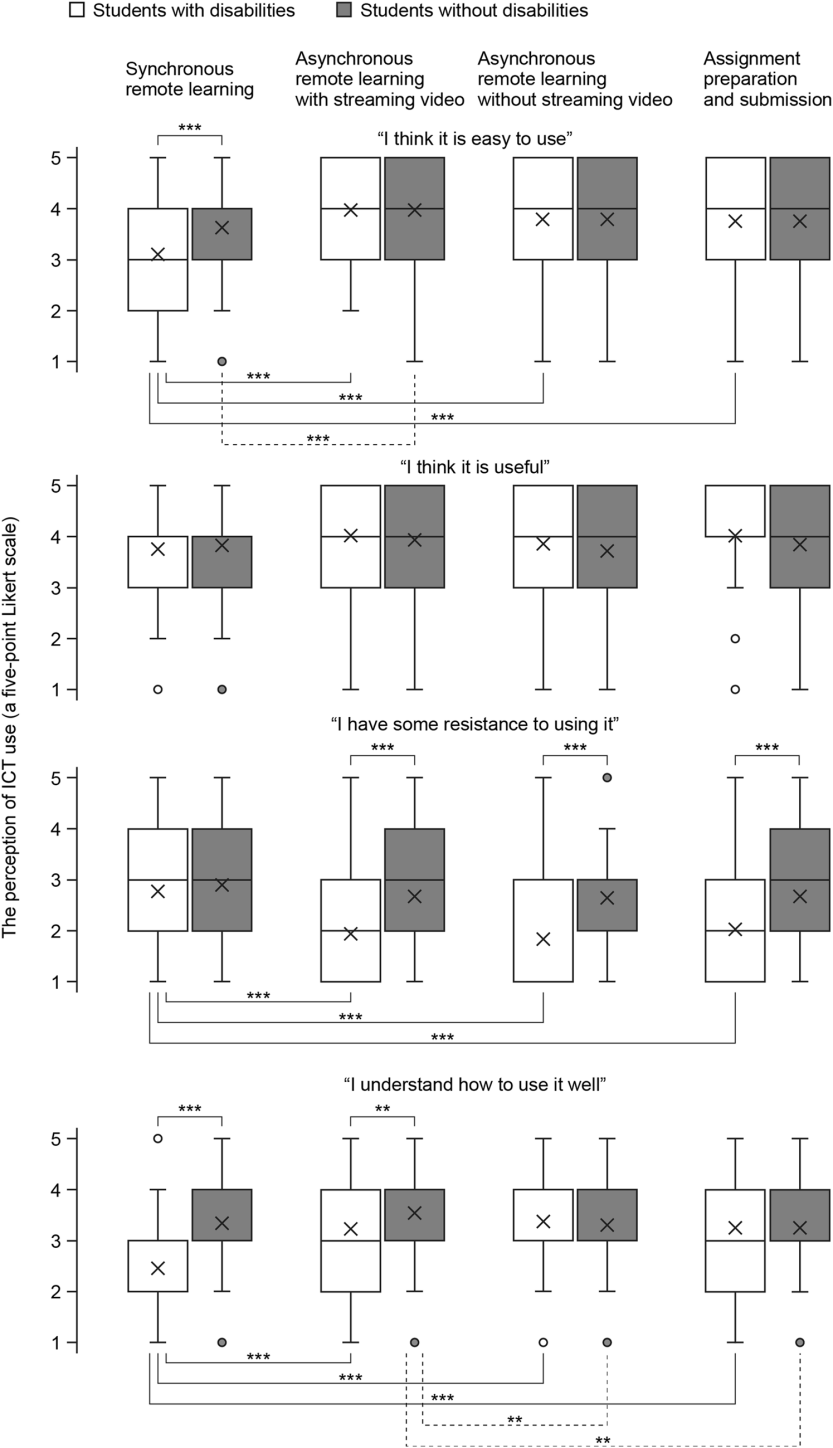
Comparison of the perceptions regarding the use of application software (before the course)

The results are shown in Fig. 2. First, we tested for simple main effects of situations regarding the perception of resistance before the course. Results showed that both the simple main effect of situations for students with disabilities (*F* (3, 705) = 16.94, *p* < 0.001) and students without disabilities (*F* (3, 705) = 2.72, *p* < 0.05) was significant. Multiple comparisons showed that students with disabilities had significantly higher perceptions of resistance before the course in synchronous remote learning than in asynchronous remote learning with streaming video (*p* < 0.001), asynchronous remote learning without streaming video (*p* < 0.001), and assignment preparation and submission (*p* < 0.001). Next, we tested for a simple main effect of disability. The results showed that there was no significant difference in the simple main effect of the presence of disability before class in synchronous remote learning (*F* (1, 235) = 0.87, *n.s.*); however, there was a significant difference in the presence of disability in asynchronous remote learning with streaming video (*F* (1, 235) = 20.30, *p* < 0.001), asynchronous remote learning without streaming video (*F* (1, 235) = 23.36, *p* < 0.001), and assignment preparation and submission (*F* (1, 235) = 14.27, *p* < 0.001). The comparison of means indicated that students with disabilities had significantly lower perceptions “I have some resistance to using” before the class in asynchronous remote learning with streaming video, asynchronous remote learning without streaming video, and assignment preparation and submission compared to students without disabilities. Hence, before taking the course, students with disabilities were generally less resistant to ICT than students without disabilities. However, only in synchronous remote learning the resistance of students with disabilities was high and at the same level as that of students without disabilities.

Second, we tested for simple main effects of situations regarding the perception of self-assessed comprehension before the course. Results showed that both the simple main effect of situations for students with disabilities (*F* (3, 702) = 18.20, *p* < 0.001) and for students without disabilities (*F* (3, 702) = 3.44, *p* < 0.05) was significant. Multiple comparisons showed that students with disabilities had a lower perception of self-assessed comprehension before the course in synchronous remote learning than in asynchronous remote learning with streaming video (*p* < 0.001), asynchronous remote learning without streaming video (*p* < 0.001), and assignment preparation and submission (*p* < 0.001). Next, we tested for a simple main effect of disability. Results showed that the simple main effect of disability in synchronous remote learning (*F* (1, 234) = 28.92, *p* < 0.001) and asynchronous remote learning with streaming video (*F* (1, 234) = 4.31, *p* < 0.05) was significant. In contrast, there were no significant differences in the simple main effects of disability on asynchronous remote learning without streaming video (*F* (1, 234) = 0.00, *n.s.*) and assignment preparation and submission (*F* (1, 234) = 0.13, *n.s.*). The comparison of the means indicated that the perception of self-assessed comprehension by students with disabilities in synchronous remote learning and asynchronous remote learning with streaming video before the course was significantly lower than that of students without disabilities. Hence, self-assessed comprehension of ICT used by students with disabilities was notably low under the condition before attending synchronous remote learning.

## Discussion

In this study, we investigated the use of ICT in distance learning in the context of the promotion of distance learning following the COVID-19 outbreak. The study aimed to identify differences in the use and perceptions of ICT during learning between students with or without disabilities enrolled in Japanese institutions of higher education based on the type of classes. In line with the study’s objectives, the following issues are discussed: ICT use by students in distance learning, perceptions of the difficulty and convenience of using ICT for students with disabilities, and future issues and limitations.

### ICT use by students in distance learning

In this study, no devices or application software were explicitly used by students with a particular type of disability. The item with the highest response regarding the use of adaptive devices and adaptive application software was “none in particular”, implying that they did not use adaptive devices or application software. This is in line with previous research findings that in recent higher education, students with disabilities tend to use mainstream technology that is not adaptive owing to accessibility improvements and other factors [22, 29]. In short, even the students with disabilities who responded “none in particular” in this study may unconsciously use standard features of general devices and application software as assistive technology. It should be noted, however, that most of the students with disabilities were students with mental health-related disabilities. Simpson et al. [30] reported that among students with disabilities enrolled in postsecondary education, students with cognitive and psychological disabilities were less likely to use assistive technology services than students with other disability types. Therefore, the bias in the types of disabilities may have overemphasized the image of students with disabilities who do not use adaptive technology. In short, although this topic should require further study, this study showed low utilization of adaptive technology.

In comparing the application software used based on disability status, Microsoft Word, Microsoft PowerPoint, Notepad, and Sticky Notes were used significantly more by students with disabilities than students without disabilities. The results suggested that these applications preferred by students with disabilities can be characterized by familiarity with their use, little change, limited use, and flexible editing. Furthermore, the reasons why students with disabilities cited Microsoft Word and Microsoft PowerPoint as the “application software that they often found particularly useful” and “ICT that they would like to continue using in the future” were found to be “familiarity with using” and “ability to edit”. The high usage rate of “Notepad” and “Sticky Notes” may be attributed to the fact that their functions do not change, which may promote their continued use by students with disabilities. In contrast, Microsoft Teams is an example of application software with various functionality. Microsoft Teams has various features, including scheduling meetings, sharing invitation links, conducting web conferences, sharing files, sharing screens, communicating via chat, and recording web conferences [31]. In addition, new features continue to be added [32]. Application software that can be continually updated and has a variety of uses is likely to be particularly unsettling for students with disabilities. In short, students with disabilities may prefer application software that does not change in function or usage.

### Perceptions of difficulty and convenience of ICT use for students with disabilities

#### Positive perceptions of ICT use for students with disabilities

Regarding device operation preference and self-rated proficiency, students with disabilities were more likely to be in the positive group for both. In contrast, previous research has suggested that students with disabilities resist computers. For example, Conti-Ramsden et al. [33] surveyed 16- to 18-year-old participants with a history of a diagnosis of a specific language disorder using scales measuring fear of computers and anxiety about using computers. The results indicated significantly higher computer anxiety among participants with disabilities. However, many have argued that today’s students are more willing to use ICTs, considering the background of growing up in an era of frequent technology use [19, 34]. Thus, resistance to technology by students with disabilities may vary from generation to generation because students with disabilities reported significantly less “difficulty” and significantly more “convenience” use of ICT in class than students without disabilities. Our findings also support the assumption that students with disabilities perceive ICT positively [29]. Fichten et al. [29] surveyed the types of technology students were asked to use in their classes during the COVID-19 pandemic and the problems they experienced when using them. They argue that because mainstream technology has a full range of accessibility features, students with disabilities and students without disabilities are likely to encounter similar difficulties. Therefore, students with disabilities experienced less difficulty in this study.

#### Pre-classroom resistance to and self-assessed comprehension of the use of applications by students with disabilities

The analysis of perceptions of the use of application software showed that students with disabilities have different perceptions before and after the course. The results before the course imply that students with disabilities tend to feel more anxious in the initial stages of using unfamiliar application software that they were less likely to use in their pre-COVID-19 situation. Regarding students’ actual use of application software before the COVID-19 pandemic, Fichten et al. [35] surveyed students with disabilities, university staff, and other professionals from 2018 to 2019. The survey reported using word processing software such as Google Documents, slide creation software such as Microsoft PowerPoint and Google Slides, and communication applications such as WhatsApp and Facebook. However, this study did not include the application software in a web conferencing system such as Zoom. Therefore, regardless of their disabilities, many students might have been unfamiliar with synchronous remote learning because it was their first experience attending the course using the web conferencing system since FY2020. This result is also related to the fact that the degree of “difficulty” of synchronous remote learning was significantly higher than in all other situations, regardless of disability. Among the students unfamiliar with the interaction class, especially students with disabilities, seemed to have increased anxiety before the course regarding resistance to and self-assessed comprehension of application software such as web conferencing systems. As discussed in Sect. 4.1, students with disabilities may have a particular preference for application software with which they are familiar. However, the reality of ICTs in education is changing [36]. Considering the changes in ICT, considerable anxiety about new application software may be a critical barrier for students with disabilities. In short, students with disabilities may be less able to keep up with developments than students without disabilities because they are more resistant and have lower self-assessed comprehension of ICTs, which they have less experience in using.

#### Improved post-course perception of students with disabilities

Before the course, students with disabilities had negative perceptions of the application software used regarding several items, including resistance to synchronous remote learning. However, after taking the course, the perceptions have changed significantly positively. Therefore, students with disabilities who had been anxious before the course were able to learn how to use the web conferencing system and its convenience by participating in the actual course. This result may address the issue discussed in Sect. 4.2.2, namely that students with disabilities tend to have high anxiety about “new application software”. More precisely, if students can learn how to use ICT and understand its convenience under conditions similar to those in actual classes, it may be possible to eliminate the anxiety of students with disabilities effectively. The assertion that creating practical opportunities for students to experience the power of ICT is the essential support required by universities has also been raised as an issue related to developing ICT use skills in teacher training programs [37]. Therefore, providing ICT use experience to learners in class situations may be one of the practical measures to promote ICT use in teaching and learning. In addition, the perceptions that changed positively in this study may be related to ICT self-efficacy. ICT self-efficacy is described as “their perceived competencies to use ICT for teaching and learning purposes, or as the confidence in their abilities to use ICT effectively in instructional practice” [37, 38]. As in the case of this research, ICT self-efficacy is measured by self-report measures. Therefore, when measuring ICT self-efficacy, we should consider whether students may overestimate or underestimate their capabilities [39]. In other words, also in this study, it is undeniable that students with disabilities may have overestimated their self-assessed comprehension of ICT. That said, increased ICT self-efficacy has the potential to act as a driving force to increase one’s knowledge and skills [39]. In summary, the negative perceptions that students with disabilities have toward new ICTs can change to positive perceptions when they complete a course that incorporates such ICTs. The positive perceptions may be helpful in that they can improve actual knowledge and skills regarding it.

### Future work and limitations

One of the limitations of this study is that it was not possible to investigate the course contents and the reasonable accommodations that the participants received. These may influence students’ degrees of difficulty and convenience in using ICT during class and their perceptions when using application software. For example, students with developmental disabilities or mental health issues may become nervous or anxious when their camera is turned on in a synchronous remote class [25]. Therefore, in classes where the instructor forces the students to turn on their cameras, some students with disabilities may feel aversion towards the web conferencing system used in the class. Second, the response rate for students with disabilities was low in this survey. In particular, it is essential to note the low response rate for students with physical disabilities and the high number of students with mental health-related disabilities; as discussed in Sect. 4.1, disability type bias may have affected the rate of adaptive technology use. Third, the response to RQ3 is based on a retrospective evaluation. The validity of the retrospective evaluation has been questioned because it is subject to recall bias [40]. Fourth, the study did not include gender in the fact sheet. Since there are various findings on the relationship between ICT use and gender among students [19], it is highly possibility that gender differences affect ICT use. Finally, we have yet to examine the relationship between ICT use and perception of ICT. Given that students with disabilities prefer application software with which they are familiar, it is likely that the improvement in perception throughout the course has an impact on ICT use. However, since the main objective of the study is to identify differences in ICT use and perception by disability and course format, we could not examine the interactive relationship between ICT use and perception. Therefore, it is necessary to examine the relationship between ICT use and the perception of students with disabilities in the future.

With regard to future work, the negative perceptions of students with disabilities before the class improved significantly after the course, demonstrating the potential for further research. If it is possible to concretize what experiences in the course led to improved perceptions, it would be possible to construct a “place to recognize usage and convenience” based on clear evidence.

## Conclusions

This study investigated the perceptions of students with and without disabilities and the characteristics of the ICT they use in remote learning promoted during the COVID-19 pandemic. Furthermore, we compared collected data quantitatively with students with and without disabilities to identify the perceptions of ICT characteristics of students with disabilities. Overall, the results show that students with disabilities have positive perceptions of ICT use, such as liking and being good at it and having low resistance. The results are unique in a survey on COVID-19 pandemic in that they reveal positive aspects of ICT use by students with disabilities. This study can further promote the use of ICT by students with disabilities by disseminating the findings that students with disabilities are positive about ICT. Furthermore, students with disabilities may prefer application software with characteristics such as “familiarity with use”, “little change”, “limited use”, and “flexible editing”. The results provide material for considering the requirements for the universal design of application software. In addition, students with disabilities were more resistant to, and their comprehension by self-assessment was lower, for using web conferencing systems before synchronous remote learning. Although students were probably anxious about using unfamiliar web conferencing systems, their anxiety tended to improve as they used the systems in class. Therefore, in anticipation of the learning environment that is likely to change rapidly with the development of ICT technology, it is suggested to emphasize the process from when students with disabilities first use ICT to the stage when they understand how to use it. For example, given the possibility that the experience of using ICT in classroom environments can be effective in improving negative perceptions, it is necessary to ensure that students with disabilities have practice opportunities similar to the classroom where they can experience how to use it and understand how convenient it is. To conclude, this study provides evidence that students with disabilities have a favorable view of ICT while at the same time suggesting conditions that make students with disabilities anxious regarding ICT and ways to alleviate this anxiety.

## Data Availability

The datasets generated during and/or analysed during the current study are available from the corresponding author on reasonable request.
